# A Case of Rheumatoid Arthritis in the Wake of Bereavement

**DOI:** 10.7759/cureus.95068

**Published:** 2025-10-21

**Authors:** Priya Nayak, Lilit Babayan, Mario Tempe, Abdul Waheed

**Affiliations:** 1 Department of Family Medicine, Creighton University East Valley Arizona, Gilbert, USA; 2 Department of Family Medicine, Creighton University School of Medicine, Phoenix, USA; 3 Department of Family Medicine, Dignity Health Medical Group, Gilbert, USA

**Keywords:** adult rheumatoid arthritis, auto immune, auto-immune, grief and bereavement, impact of stress, life stress, rheumatoid arthritis

## Abstract

Psychological stress, including bereavement and depression, can have immunological consequences that promote the development of autoimmune disease. Activation of the hypothalamic-pituitary axis (HPA) and sympathetic nervous system are potential mechanisms for crosstalk between emotional stress and immune system function. Studies have shown increased proinflammatory cytokines in individuals who experience major life stressors; these individuals are also at increased risk for developing autoimmune diseases, including rheumatoid arthritis. In this report, we present the case of a woman who presented to primary care after the sudden death of her son. She was found to be prediabetic with hyperlipidemia and, within several months of the acute psychological stressor, was diagnosed with rheumatoid arthritis (RA). Altogether, this new diagnosis of RA was likely triggered by grief, with a background of potential predisposing factors. This case illustrates a close temporal relationship between an acute stressor and onset of autoimmune disease. It underscores the importance of integrated care and psychosocial considerations in the evaluation and management of autoimmune diseases.

## Introduction

The reciprocal relationship between psychological stress and immune system dysfunction has important implications for the development of autoimmune disease [1]. Rheumatoid arthritis (RA), an autoimmune disease of progressive polyarticular and systemic inflammation that affects 0.5%-1% of the US population, arises through a complex interplay of genetic susceptibility and environmental exposures. Identical twin studies show disease concordance for RA to be only 12%-15%, indicating a central role for environmental triggers in the development of disease [1]. Recent estimates indicate a global prevalence on 0.46% for RA [2]. Environmental factors that favor the development of RA include smoking, vitamin D deficiency, obesity, and microbiome perturbations. In addition, associations between psychological stress and autoimmunity have been recognized as potential drivers of disease [3].

Bereavement is a major psychological stressor that follows a highly individualized course depending on cultural norms, social support, a patient’s history and character traits, and specific life situation. Thus there is debate surrounding what constitutes normal grief versus a disorder [4,5]. Nevertheless, grief can have significant overlap with the symptoms of major depression, including depressed mood, loss of interest, insomnia, and lack of concentration, and therefore constitutes a significant psychological stress, with accompanying immunological consequences [6].

Psychological stress can activate the hypothalamic-pituitary-adrenal (HPA) axis and sympathetic nervous system. The HPA axis comprises three components of the endocrine system that secrete hormones as part of the stress response. Similarly, the sympathetic immune system is activated in “fight or flight” responses. This activation alters levels of glucocorticoids and catecholamines, which in turn impacts immune regulation and promotes proinflammatory cytokine activity [7]. Several studies - including prospective longitudinal observational studies, case reports, and meta-analyses - have shown that patients experiencing major life stressors demonstrate elevated markers of inflammation and may be at increased risk for autoimmune conditions, including systemic lupus erythematosus, inflammatory bowel disease, and rheumatoid arthritis [8-10]. A meta-analysis by Segerstrom and Miller demonstrated that acute and chronic stressors can dysregulate immune function through the upregulation of proinflammatory cytokines [11]. Indeed spousal bereavement has been linked to a pro-inflammatory state, which may predispose the development of disease, including cardiovascular disease and autoimmunity [12].

While the contribution of stress to overall development and disease activity flares of RA is relatively well-characterized, less is known about the relationship between an acute stressful trigger and incitement of autoimmunity. In this report, we present the case of a previously healthy woman who developed RA within a few months of experiencing the sudden death of her son. Our report adds to the literature on stress-induced autoimmunity and highlights the need to consider recent emotional trauma as a potential contributor to disease onset - much like Takotsubo syndrome in cardiology [13]. This case encourages consideration of psychosocial factors in autoimmune disease evaluation, supporting more integrated models of patient care.

## Case presentation

A 47-year-old female patient with no prior significant past medical history presented to the clinic on September 5, 2024, to establish care and to request paperwork for work leave due to depression under the Family and Medical Leave Act (FMLA). About two months earlier, the patient’s 27-year-old son had passed away in his sleep suddenly and of unclear cause. The patient lived with three of her five children and one grandchild, including the son who passed away. The death left the family “devastated.” Since then, she reported depressed mood and poor sleep. At the initial visit, her Patient Health Questionnaire-9 (PHQ-9) score was 19. Her physical exam was notable for a body mass index (BMI) of 31 and depressed mood and affect. She did not smoke and did not take any medications.

The patient was diagnosed with a major depressive episode and transient insomnia, both attributed secondary to grief. Work release paperwork was provided, and treatment options, including pharmacotherapy and behavioral health counseling, were discussed. The patient opted for counseling and a referral was placed for behavioral health. In addition, labs were ordered to rule out other potential causes of depressed mood, as well as for health screening (complete blood count (CBC), comprehensive metabolic panel (CMP), thyroid stimulating hormone (TSH), lipid panel, hemoglobin A1C) (Table 1).

**Table 1:** Initial Work-Up and Screening Labs Complete blood count, comprehensive metabolic panel, thyroid stimulating hormone, hemoglobin A1C were obtained as an initial work-up for the patient’s depressive symptoms. In addition, a lipid panel was obtained as part of routine screening for the patient’s age and elevated BMI. These labs were notable for modestly increased values of multiple measures of chronic metabolic dysregulation (values in bold), including mixed hyperlipidemia, pre-diabetic range HbA1C, and mildly elevated uric acid. A mildly decreased mean corpuscular volume (MCV) with normal hemoglobin is of subclinical significance but could perhaps be indicative of an underlying inflammatory condition or mild iron deficiency. These factors could contribute to the development of rheumatoid arthritis in this patient. Bolded values are outside of normal ranges

Lab	Unit	Most recent	Normal Range
Complete blood count			
White blood cells	'000/ul	8.2	4.5-13.5
Red blood cells	million/uL	4.84	4.00-5.40
Hemoglobin	g/dL	12.6	11.5-16.0
Hematocrit	%	38.0%	37.0-47.0
Mean corpuscular volume	fL	78.6	80-100
Mean corpuscular hemoglobin	pg	26	27.0-34.0
Mean corpuscular hemoglobin concentration	g/dL	33.1	32.0-37.0
Red cell distribution width	%	15.2%	11.5-16.0
Platelets	'000/uL	296	130-400
Mean platelet volume	fL	8.1	7.5-11.5
Neutrophils	%	62.6%	35.0-80.0
Lymphocytes	%	23.4%	10-55
Monocytes	%	10.2%	0-15
Eosinophils	%	2.8%	0-9
Basophils	%	1.0%	0-3
Complete metabolic panel			
Sodium	mmol/L	139	136-145
Potassium	mmol/L	4.4	3.5-5.1
Chloride	mmol/L	104	98-107
Carbon dioxide (CO_2_)	mmol/L	22	20-27
Anion gap		17	10-20
Glucose	mg/dL	97	70-105
Blood urea nitrogen		10	9-21
Creatinine	mg/dL	0.69	0.57-1.25
Estimated glomerular filtration rate, creatinine	mL/min/1.73m^2^	108	≥90
Calcium	mg/dL	9.2	8.4-10.2
Uric acid	mg/dL	6.3	2.6-6.0
Protein, total	g/dL	7.2	6.0-8.3
Albumin	g/dL	4.2	3.5-5.0
Globulin	g/dL	3	2.3-3.5
Albumin/globulin Ratio		1.4	0.8-1.6
Bilirubin, total	mg/dL	1.2	0.2-1.2
Alanine transaminase	Units/L	26	6-55
Aspartate aminotransferase	Units/L	20	5-34
Alkaline phosphatase	Units/L	105	40-150
Lipid panel			
Total cholesterol	mg/dL	218	0-200
High-density lipoprotein (HDL)	mg/dL	46	≥40
Very low-density lipoprotein	mg/dL	22	0-40
Cholesterol/HDL		5	≤5
Low-density lipoprotein-calculated	mg/dL	150	≤99
Non-HDL	mg/dL	172	≤130
Triglycerides	mg/dL	108	0-150
Special chemistry			
Hemoglobin A1C	%	5.7%	≤5.6
Thyroid stimulating hormone	mcIU/ mL	2.040	0.350-4.940

At the following visit on November 7, 2024, the patient had not yet been able to see a grief counselor. The labs were notable for a prediabetic hemoglobin A1C (5.7%) and mixed hyperlipidemia, for which atorvastatin 10 mg daily was started.

The patient returned to the clinic on March 6, 2025, to follow-up for hyperlipidemia; she had not yet been able to attend grief counseling. Furthermore, she reported that for the past six to eight weeks, she had been having intermittent knuckle pain, swelling, and redness, with about 30 minutes of daily morning stiffness. Her physical exam was notable for stiffness and tenderness of the metacarpophalangeal (MCP) joints, as well as restriction of movement of the right shoulder with joint line tenderness. Meloxicam 15 mg daily was started and rheumatologic labs were drawn.

At the follow-up appointment on March 27, 2025, the patient reported slight symptomatic relief with meloxicam, however her labs were notable for elevated high-sensitivity C-reactive protein (hsCRP) (4.38 mg/L), rheumatoid factor (173.5 IU/mL), and anti-cyclic citrullinate peptide (anti-CCP) IgG/IgA (172 U/mL). Rheumatoid factor and anti-CCP are both auto-antibodies that are produced abnormally and that target normal tissues to produce the manifestations of rheumatoid arthritis. Indeed, higher rheumatoid factor (RF) levels are predictive of more active disease and extra-articular manifestations [2]. Her joint involvement now included multiple MCP joints in both hands, right wrist, elbow, and shoulder, and left knee. A diagnosis of rheumatoid arthritis was made; the temporal relationship of grief may have played a role in the manifestation of symptoms and elevation of rheumatological markers. Hydroxychloroquine 400 mg daily and a 30-day prednisone taper with starting dose 20 mg was started. The patient was referred to rheumatology (Table 2). 

**Table 2:** Inflammatory and Autoimmunity Labs Inflammatory and auto-immunity labs were obtained to evaluate patient’s polyarticular pain and swelling in multiple small and large joints. These physical exam findings, in conjunction with the elevated rheumatoid factor (RF) and anti-CCP (anti-cyclic citrullinate peptide) antibodies make a diagnosis of rheumatoid arthritis most likely. Bolded values are outside of normal ranges.

Lab	Unit	Most recent	Normal Range
Inflammatory markers			
Erythrocyte sedimentation rate	mm/h	9	0-19
C-reactive protein (CRP)	mg/dL	0.16	0.10-0.90
CRP high sensitivity	mg/dL	4.38	≤5.0
Autoimmunity			
Rheumatoid factor	IU	173.5	≤30
Anti-nuclear antibody, immunoglobulin G (IgG)	IU	None Detected	
Cyclic citrullinated peptide IgG/IgA	IU	172	<20

At a subsequent visit on April 24, 2025, the patient had consulted rheumatology in the interim; methotrexate 10 mg per week was prescribed. The patient reported lessening of pain in her hands but continued to have pain and stiffness in her large joints. She had still not had an initial visit with grief counseling. Potential treatment options for grief and depression were discussed with the patient, including starting a selective serotonin reuptake inhibitor (SSRI), but the patient did not opt for pharmacological treatment at that time. She was encouraged to make an appointment with the grief counselor. The patient was in clinical remission on methotrexate at three months follow-up.

## Discussion

In this case report, a 47-year-old female patient presented to primary care with severe depressive symptoms about two months after the sudden death of her adult son. About three months later (five months after the death of her son), she began to have intermittent joint pain and morning stiffness, which was confirmed to be seropositive rheumatoid arthritis. Simultaneously, the patient had an elevated BMI and was found to have pre-diabetes and mixed hyperlipidemia. Taken together with her age and gender risk factors, this presents a picture of a new diagnosis of RA following a major depressive episode due to grief, in the context of preexisting chronic disease (metabolic syndrome).

Comorbidity of psychiatric and autoimmune disorders have been well-documented in the literature. Chronic conditions, including depression, post-traumatic stress disorder (PTSD), and adverse childhood events, can promote the development of autoimmune disease as well as worsen the course of pre-existing disease [10,14]. At the same time, there are unique barriers to care for grief and other mental health issues that may make optimal care more difficult. These include lack of access, lack of information about how to access care, less knowledge about mental health, and hesitancy to access behavioral health services. The bidirectional occurrence of depression and autoimmune disease indicates that there could be common underlying risk factors that predispose the development of both conditions [15]. By contrast, less is known about the role of an acute psychological stressor in the potential incitement of autoimmune disease. While a causal relationship cannot be determined from this report, this report adds to the literature with a case of a close temporal relationship between an acute episode of severe emotional stress and new onset of rheumatoid arthritis.

The multifactorial nature of both chronic predisposing factors as well as potential acute triggers is highlighted. While potential genetic and chronic stress contributors are unknown in this patient, her gender, age, and obesity could be chronic predisposing factors. The patient’s acute depressive episode is a potential trigger for her RA; other potential triggers, including other environmental exposures or the patient’s microbiome are unknown. Further clinical and basic science studies are required to tease apart the roles and potential synergies of the various chronic and acute factors. For example, immunological and hormonal perturbations associated with chronic and acute psychiatric conditions are not well characterized, especially in the context of autoimmune disease.

Mirroring the multi-factorial contributors to the development of rheumatoid arthritis, the treatment in this case was also multi-pronged: The patient was aided with obtaining medical leave, referred for grief counseling, and was provided the option of pharmacological treatment for depressive symptoms. The patient’s insomnia was addressed. She was started on a statin for hyperlipidemia. She was referred to rheumatology for immunosuppressive treatment and continued to have close primary care follow up. As such, the case underscores the crucial role of team-based care in assessing and managing RA.

## Conclusions

We present the case of a 47-year-old woman who was diagnosed with rheumatoid arthritis five months after the sudden death of her son. While the bi-directional nature of psychological stress and autoimmunity is well-documented, this report presents a unique close temporal association between an acute stressor and symptom onset. While there is no formal recommendation for screening for autoimmunity, clinicians are encouraged to have a higher index of suspicion and close follow-up in bereaved patients. Importantly, we highlight the central role of integrated care in the management of medical and psychological aspects of rheumatoid arthritis.
